# Prevalence estimates of multimorbidity: a comparative study of two sources

**DOI:** 10.1186/1472-6963-10-111

**Published:** 2010-05-06

**Authors:** Martin Fortin, Catherine Hudon, Jeannie Haggerty, Marjan van den Akker, José Almirall

**Affiliations:** 1 Department of Family Medicine, Sherbrooke University, Sherbrooke, Québec, Canada; 2 Department of Community Sciences, Sherbrooke University, Sherbrooke, Québec, Canada; 3 Department of General Practice, Care and Public Health Research Institute, Maastricht University, Maastricht, The Netherlands

## Abstract

**Background:**

Published prevalence studies on multimorbidity present diverse data collection methods, sources of data, targeted age groups, diagnoses considered and study populations, making the comparability of prevalence estimates questionable. The objective of this study was to compare prevalence estimates of multimorbidity derived from two sources and to examine the impact of the number of diagnoses considered in the measurement of multimorbidity.

**Methods:**

Prevalence of multimorbidity was estimated in adults over 25 years of age from two separate Canadian studies: a 2005 survey of 26,000 respondents randomly selected from the general population and a 2003 study of 980 patients from 21 family practices. We estimated the prevalence of multimorbidity based on the co-occurrence of ≥ 2 and ≥ 3 diseases of the seven diseases listed in the general population survey. For primary care patients, we also estimated multimorbidity prevalence using an open list of chronic diseases.

**Results:**

Prevalence estimates were considerably higher for each age group in the primary care sample than in the general population. For primary care patients, the number of chronic diseases considered for estimates resulted in large differences, especially in younger age groups. The prevalence of multimorbidity increased with age in both study populations.

**Conclusions:**

The prevalence of multimorbidity was substantially lower when estimated in a general population than in a family practice-based sample and was higher when the number of conditions considered increased.

## Background

As a result of different factors, including aging populations and advances in medical care and public health policy, a growing proportion of patients present multiple coexistent diseases, or multimorbidity [1]. To estimate the magnitude of this problem, studies about the prevalence of multimorbidity have been conducted in different parts of the world: in Europe [1-5], the Middle East [6], Australia [7], the United States [8-10], and Canada [11-13]. These studies present diverse sources of data (e.g., questionnaires, chart reviews, administrative data), data collection methods, targeted age groups, diagnoses considered and study populations, making the comparability of prevalence estimates questionable. Some studies [5,6,8-11] focused on data from samples of the general population; others [1-3,7,13] from primary care practices. In a recent study [4] on the prevalence of multimorbidity in four different settings (population-based, general practice, hospital and nursing home), the authors concluded that the study population in research on the prevalence of multimorbidity required special attention. Their study, however, was limited to people ≥ 55 years of age, and the diseases considered for prevalence estimates of multimorbidity varied in the different study populations. Furthermore, information about all morbidities (complaints, diseases and disorders) was collected from medical charts for patients in general practice and from a limited list of chronic conditions for the general population [4]. We believe that differences among the diagnoses considered could have influenced estimates of the prevalence of multimorbidity. Analysis of how the prevalence of multimorbidity in the general population is reflected in clinical practice has not yet been done. To make a valid comparison of prevalence estimates, data should be obtained simultaneously or within a short time frame from both general and primary care populations. In addition, the medical conditions taken into account and the operational definition of multimorbidity used should be consistent. Data from the general population, although useful for a comparison of different geographical areas, provide an incomplete picture of where chronic diseases are dealt with, for example, in the offices of family doctors.

A recent publication [14] in which prevalence estimates of multimorbidity were calculated using data obtained in 2005 from the general population of the province of Quebec, Canada, allowed us to make a comparison with prevalence estimates of multimorbidity using data from a 2003 family practice-based study [13] in the same province. The aim of the current study was to compare age- and sex-specific estimates of the prevalence of multimorbidity derived from these two populations, and to examine the impact of a variation in the number of different diagnoses considered on prevalence estimates within the practice-based population.

## Methods

The prevalence of multimorbidity in the general population of Quebec comes from a Quebec publication [14] based on data from the Canadian Community Health Survey (CCHS). The CCHS is a series of general surveys that Statistics Canada http://www.statcan.gc.ca has carried out since 2000. Approximately 132,000 randomly-selected Canadians (26,000 from Quebec) 12 years and older participate, either in person or by telephone, in a 45-minute computer-assisted interview. For the current study, we used data collected in the 2005 CCHS [14] on the self-reported presence of seven diseases: arthritis, cancer, diabetes mellitus, hypertension, heart disease, obstructive lung disease and psychiatric problems. We limited calculations to results from participants aged 25 years and over to permit comparisons.

We obtained family practice-based data from a study conducted by our research group in the Saguenay region of Quebec in 2003 [13]. In this study, trained research staff extracted diagnoses of chronic diseases from patients' medical charts. Details of the methods and sampling strategies used in this study are described elsewhere [13]. The regional ethics committee approved the original Saguenay study. In brief, 980 (90%) of 1085 consecutive adult patients solicited during successive consultation periods from 21 family physicians' practices consented to participate.

For the current study, we limited chronic diseases to the same seven diseases included in the CCHS. We also calculated estimates of the prevalence of multimorbidity in the family-practice patients with an open list of chronic diseases, i.e., all the diseases that might have appeared in the patients' medical records.

We retained the two operational definitions of multimorbidity used in the CCHS report [14]; that is, we defined multimorbidity as (1) ≥ 2 diseases and (2) ≥ 3 diseases. Both definitions were previously used in several other studies [13-17]. Disease complications were considered as distinct diseases. For the comparison of prevalence data between the two studies [13,14], we compared multimorbidity data for participants ≥ 25 years of age only because these data were available from both the Saguenay [13] and the CCHS [14] studies. Subjects were grouped by age as follows: 25-44 yr, 45-64 yr, 65-79 yr, and 80 yr and over. The analysis was done at the patient level only, as clustering of patients by physician in the Saguenay study was negligible.

We estimated age-specific and sex-specific prevalence, without adjustment for the cluster sample study design and subsequently calculated age-standardized prevalence using the general population as a reference. To determine the significance of different prevalence estimates we calculated Fisher's 95% confidence intervals looking for overlap and also compared them graphically.

## Results

Table 1 shows demographic information for the two samples. Because the age distribution of the two populations was different, age-specific analyses of multimorbidity were done. Because age distribution by sex was not available from the CCHS, the sex distributions included data from subjects ≥ 12 years of age in the general population and subjects ≥ 25 years of age in the family practice-based population. Compared with the general population, the prevalence of ≥ 2 chronic diseases in the family practice-based population was 5.0 times higher in men and 3.5 times higher in women; prevalence of ≥ 3 diseases was 9.0 times and 4.5 times higher, respectively. More women than men were found with multimorbidity in the general population, whereas the reverse was found in the practice-based population. In the general population, 10.1% (95% confidence interval [CI]: 10.0-10.1) of men and 13.3% (CI: 13.2-13.3) of women had ≥ 2 diseases; in the practice-based population, results were 51.9% (CI: 46.3-57.5) of men and 46.1% (CI: 42.2-46.1) of women. The proportion of men with ≥ 3 diseases in the general population was 3.4% (CI: 3.38-3.42); the proportion of women was 4.5% (CI: 4.4-4.5). The proportions were 30.9% (CI: 25.9-36.3) of men and 20.2% (CI: 17.2-23.4) of women in the practice-based population. Note that no confidence intervals overlap. Because analyses by sex for specific age groups were not provided for the general population study, we did not calculate age- and sex-standardized rates for the family practice-based population.

**Table 1:** Demographic information

	No. (%)	
	
Group	Estimated population of Quebec (*N *= 6,447,800)	Saguenay family practice-based sample (*N *= 938)
Males*	3,171,500 (49.2)	315 (33.6)
Females*	3,276,300 (50.8)	623 (66.4)
25-44 y	2,147,800 (33.3)	179 (19.1)
45-64 y	2,088,100 (32.4)	407 (43.4)
65-79 y	771,700 (12.0)	239 (25.5)
≥80 y	187,200 (2.9)	52 (5.5)

*Age of Quebec estimated population: ≥12 years; age of Saguenay sample: ≥25 years.

**Figure 1:**
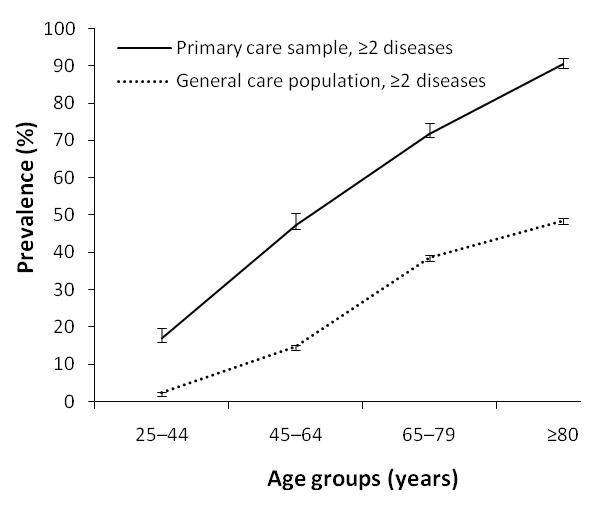
**Age-specific prevalence of multimorbidity and 95% confidence intervals (error bars) for those with ≥ 2 diseases**.

The age-specific overall prevalence of ≥ 2 diseases for both study populations is shown in Figure 1. The prevalence in each age group is substantially higher in the practice-based than in the general population. Also, the rates of increase in the prevalence of morbidity (≥ 2 diseases) with age differed between the two study populations (approximately 1.3%/yr in the practice-based, and 0.8%/yr in the general population). The age-standardized prevalence of ≥ 2 diseases (25 yr and over; reference: general population) was 32.3% in the practice-based and 11.6% in the general population: a 2.8:1 ratio.

**Figure 2:**
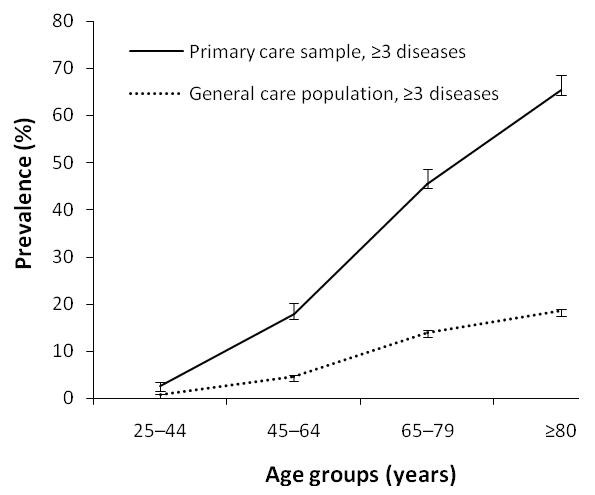
**Age-specific prevalence of multimorbidity and 95% confidence intervals (error bars) for those with ≥ 3 diseases**.

In Figure 2, the age-specific overall prevalence of ≥ 3 diseases for both study populations is shown. As with the previous operational definition, the prevalence in each age group is substantially higher in the practice-based than in the general population and the differential in the annualized increase in the prevalence of ≥ 3 was even more pronounced than for ≥ 2 diseases: 1.1%/yr vs. 0.3%/yr. The age standardized prevalence of ≥ 3 diseases (25 yr and over; reference: general population) was 14.0% in the practice-based population and 3.9% in the general population: a 3.6:1 ratio.

**Figure 3:**
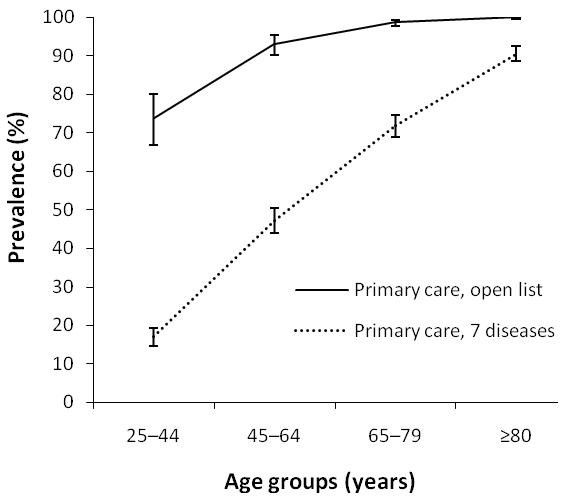
**Prevalence of multimorbidity (as defined by the presence of ≥ 2 diseases) and 95% confidence intervals (error bars) estimated with a list of seven diseases and with an open list**.

Figure 3 compares prevalence estimates of multimorbidity (defined as ≥ 2 diseases) based on the list of seven conditions with those based on an open list of chronic diseases for the family practice-based group of patients. Differences were marked, especially for younger age groups. Multimorbidity prevalence estimates for the 25-44 year group were 17.1% when based on the list of seven conditions, and 73.9% when based on an open list of chronic diseases in the practice-based group. For the 45-64 year group, prevalence estimates were of 47.3% and 93.1%, respectively.

## Discussion

Results of the current study suggest that estimates of the prevalence of multimorbidity based on a simple count of diseases for the general population are not equivalent to those for family practice-based populations. Sex- and age-specific prevalence estimates of multimorbidity as well as age-standardized prevalence estimates are substantially higher in the family practice-based than in the general population. The nature of the study population is, therefore, a major factor in the accurate interpretation of studies of the prevalence of multimorbidity. In the current study, for patients with ≥ 2 chronic diseases -- the classical definition of multimorbidity -- the difference in prevalence estimates of multimorbidity for the family practice-based and general care studies reached 40%. Based on age-standardized estimates, the prevalence was approximately three times higher for the primary care-based population. Differences were even more marked for patients with ≥ 3 chronic diseases.

Prevalence estimates of multimorbidity in the general population are important for reporting about the health status of the population. However, the results of the current study suggest that the clinical burden of multimorbidity is higher in family practice than would be expected from data collected for the general population, highlighting the importance of having prevalence estimates at the practice level, and the development and implementation of practice-based epidemiological research. Because of the large percentage of patients with multimorbidity, including geriatric patients, in the primary care population, primary care settings must be strengthened quantitatively and qualitatively to keep pace with this growing problem.

The operational definition considered in prevalence studies about multimorbidity is the second most important concern. In the current study, we found that the greater the number of diagnoses included, the higher the prevalence estimates of multimorbidity. Using the same classical definition of multimorbidity and the same age groups, but varying the number of diagnoses considered (a list of 7 conditions vs. an open list) to compare prevalence estimates for the same family practice-based population, we found large differences in these estimates across all age groups (Fig. 3).

Moreover, not only the number of diseases, but also the way they are documented is important. Prevalence estimates of multimorbidity calculated with a different definition in a Netherlands study [2] were well below those of the current study calculated with an open list of diagnoses. The Netherlands study [2] analyzed data from a database of 60,857 patients from a registration network of family practices in the Netherlands [2] to estimate the prevalence of the co-occurrence of ≥ 2 "active health problems". Health problems, based on ICPC codes related to rubrics, were defined as "active" if identified by the general practitioner or the patient, as reflected in current treatment, subsequent diagnostic investigations, disease monitoring, or the known progressive course of a disease. Prevalence of the co-occurrence of ≥ 2 active health problems for patients 20-39 years of age was 16.0% for men and 18.8% for women; for those 40-59 years of age, 33.6% for men and 35.9% for women; for those 60-79 years of age, 60.9% for men and 64.9% for women; for those ≥ 80 years of age 74.2% for men and 79.9% for women. Compared with the study in Saguenay, these different results may represent real differences in the prevalence of multimorbidity or may be the consequence, at least in part, of the way the diseases were documented in each study. Sampling also contributes to the difference. The Saguenay study recruited patients attending the practice. This could bring out a higher proportion of patients with complex needs as they consult more often and therefore have a higher chance of being selected. At the same time, this provides us with a good estimate of the burden at the practice level. On the other hand, the Netherlands study included all patients from the register (including those consulting less often). This could explain part of the difference. Furthermore, including complications of previous conditions could result in a higher count of diseases. For example, diabetes complicated by renal failure and neuropathy would have counted as three separate occurrences in the Saguenay study thus contributing to the higher numbers.

Many prevalence studies use a limited list of chronic conditions [4,6,7,9,11,12]; however, not including frequent conditions could affect prevalence results. Similarly, the inclusion of medical conditions considered as risk factors is controversial. For example, hyperlipidemia and obesity are two common conditions frequently omitted from prevalence studies [4,6,12]. The real requirement for medical treatment of such conditions makes a strong argument for their inclusion in the count. Limiting the number of diagnoses considered when defining multimorbidity is of special concern because of the great heterogeneity in disease burden observed among patients with chronic conditions. Ideally, studies about the prevalence of multimorbidity should be based on a standard list of chronic conditions that include at a minimum the most frequent diagnoses. From their review of the literature, Bayliss and colleagues [18] compiled a list of 24 health conditions most frequently assessed for the measurement of co-morbidity to develop an instrument for the assessment of disease burden. Such a list could be a good reference point for estimating the prevalence of multimorbidity in different populations. If the International Classification of Primary Care-Version 2 (ICPC-2) is used, a good reference point for estimating the prevalence of multimorbidity could be the list of chronic conditions designed by O'Halloran and colleagues[19], based on the ICPC-2 (list available at http://www.fmrc.org.au/Download/DefiningChronicConditions.pdf). The list, although designed to identify chronic conditions managed in Australian general practice, could be used in other settings.

According to the present study, in the general population, there were more women than men with multimorbidity, but more men than women with multimorbidity are seen in primary care. In the general practice population, prevalence rates tend to be higher among men in the younger age groups [13]. Differences in the severity of disease may lead to a different pattern of consultation or to differences in the timing of seeking medical attention [20]. In general, other population-based studies [5,6,9] have shown a higher prevalence of multimorbidity in women. For general practice, some studies [2,3] reported an age-specific prevalence that tended to be higher for younger men and higher for older women. Another study [7] reported no difference in the prevalence of multiple diseases between sexes in general practice. However, diseases were classified according to the Cumulative Illness Rating Scale (CIRS) morbidity domains and multimorbidity was defined as presence of morbidity in two or more domains rather than individual diseases. It could have contributed to the lack of differences among gender groups as diseases within the same CIRS domain would count only for one.

The strength of the current study lies in its comparison of two different sources of data that were collected within a relatively short time span, thus validating the comparison. When we look at the relatively slow rate of the incidence of chronic diseases and their long duration, the difference of two years between the two studies' data collections seems negligible for the comparison of two different populations.

This study has limitations. The comparison of prevalence estimates by sex was limited by the different age spans of the study populations (Table 1). Prevalence estimates in the current study may have been affected by the different methods of collecting the data in the two studies compared. Another limitation is that we estimated age-specific and sex-specific prevalence without adjustment for the cluster sample study design, however, it is unlikely that this has affected the findings in terms of the differences in results from the two methods because of the large differences found. In the 2005 study, trained research staff extracted diagnoses of medical conditions from patients' medical charts, making its data collection different from the self-report method used in the CCHS. Because several studies [21-25] have suggested that self-reported and record-based estimations of multimorbidity provide similar results, we considered that a comparison between these two studies was valid. However, other studies [26-28] have reported differences. The questionnaire used for the general population group is susceptible to a self-declaration bias; patients may underreport diagnoses of less importance to them or that they do not recall [27]. However, with the exception of psychiatric diseases, questions about the presence of diseases were specific in the CCHS [14] and facilitated recall. Conversely, using medical records alone may result in an underestimation of some symptom-based conditions [26]. The direction of the bias with different sources of data could go either way. However, any bias that might have been introduced in the current study is unlikely to affect the robustness of the conclusions, given the magnitude of the most important differences.

## Conclusions

Prevalence estimates of multimorbidity for the family practice-based population were higher than those for the general population, in both men and women. Age-specific prevalence estimates were also higher for all age groups in the family practice population and had a higher rate of increase with age. Based on age-standardized estimates and a classical definition of multimorbidity, the prevalence in the practice-based population was three times higher. When the number of chronic conditions considered increased, the prevalence estimate of multimorbidity was higher. The use of a limited list of chronic diseases may introduce an important bias in the prevalence estimates of multimorbidity. Reporting prevalence estimates should always specify the reference population, the method of estimation, and the data source to allow comparison and accurate interpretation of prevalence studies about multimorbidity. The clinical burden on family practice was higher than expected from the data collected for the general population, highlighting the importance of prevalence estimates of multimorbidity at the practice level.

## Competing interests

The authors declare that they have no competing interests.

## Authors' contributions

MF participated in the conception and design of the study, supervised data collection and analysis and drafted the manuscript. CH participated in the design of the study and critically reviewed the manuscript. JH participated in the design of the study, supervised the statistical analysis and helped draft the manuscript. MvdA participated in data analysis and critically reviewed the manuscript. JA participated in the data analysis and helped draft the manuscript. All authors gave their final approval of the version of the manuscript submitted for publication.

## Pre-publication history

The pre-publication history for this paper can be accessed here:

http://www.biomedcentral.com/1472-6963/10/111/prepub

## References

[B1] Uijen AA Lisdonk EH van de Multimorbidity in primary care: prevalence and trend over the last 20 years Eur J Gen Pract 2008 1 28 32 10.1080/13814780802436093 18949641

[B2] Akker M van den Buntinx F Metsemakers JF Roos S Knottnerus JA Multimorbidity in general practice: prevalence, incidence, and determinants of co-occurring chronic and recurrent diseases J Clin Epidemiol 1998 51 367 375 10.1016/S0895-4356(97)00306-5 9619963

[B3] Metsemakers JF Hoppener P Knottnerus JA Kocken RJ Limonard CB Computerized health information in The Netherlands: a registration network of family practices Br J Gen Pract 1992 42 102 106 1493025 PMC1371993

[B4] Schram MT Frijters D Lisdonk EH van de Ploemacher J de Craen AJ de Waal MW van Rooij FJ Heeringa J Hofman A Deeg DJ Schellevis FG Setting and registry characteristics affect the prevalence and nature of multimorbidity in the elderly J Clin Epidemiol 2008 61 1104 1112 10.1016/j.jclinepi.2007.11.021 18538993

[B5] Marengoni A Winblad B Karp A Fratiglioni L Prevalence of chronic diseases and multimorbidity among the elderly population in Sweden Am J Public Health 2008 98 1198 1200 10.2105/AJPH.2007.121137 18511722 PMC2424077

[B6] Fuchs Z Blumstein T Novikov I Walter-Ginzburg A Lyanders M Gindin J Habot B Modan B Morbidity, comorbidity, and their association with disability among community-dwelling oldest-old in Israel J Gerontol A Biol Sci Med Sci 1998 53 M447 455 9823749 10.1093/gerona/53a.6.m447

[B7] Britt HC Harrison CM Miller GC Knox SA Prevalence and patterns of multimorbidity in Australia Med J Aust 2008 189 72 77 18637770 10.5694/j.1326-5377.2008.tb01919.x

[B8] Wolff JL Starfield B Anderson G Prevalence, expenditures, and complications of multiple chronic conditions in the elderly Arch Intern Med 2002 162 2269 2276 10.1001/archinte.162.20.2269 12418941

[B9] Guralnik JM Assessing the impact of comorbidity in the older population Ann Epidemiol 1996 6 376 380 10.1016/S1047-2797(96)00060-9 8915467

[B10] Hoffman C Rice D Sung HY Persons with chronic conditions. Their prevalence and costs Jama 1996 276 1473 1479 10.1001/jama.276.18.1473 8903258

[B11] Daveluy C Pica L Audet N Courtemanche R Lapointe F Enquête sociale et de santé 1998 2000 2 Québec: Institut de la statistique du Québec

[B12] Rapoport J Jacobs P Bell NR Klarenbach S Refining the measurement of the economic burden of chronic diseases in Canada Chronic Dis Can 2004 25 13 21 15298484

[B13] Fortin M Bravo G Hudon C Vanasse A Lapointe L Prevalence of multimorbidity among adults seen in family practice Ann Fam Med 2005 3 223 228 10.1370/afm.272 15928225 PMC1466875

[B14] Cazale L Dumitru V Chronic diseases in Quebec: some striking facts [French] Zoom Santé 2008 March 1 4

[B15] Deuse T Detter C Samuel V Boehm DH Reichenspurner H Reichart B Early and midterm results after coronary artery bypass grafting with and without cardiopulmonary bypass: Which patient population benefits the most? Heart Surgery Forum 2003 6 77 83 12716586 10.1532/hsf.708

[B16] Kruse-Losler B Langer E Reich A Joos U Kleinheinz J Score system for elective tracheotomy in major head and neck tumour surgery Acta Anaesthesiologica Scandinavica 2005 49 654 659 10.1111/j.1399-6576.2005.00655.x 15836679

[B17] Cesari M Onder G Russo A Zamboni V Barillaro C Ferrucci L Pahor M Bernabei R Landi F Comorbidity and physical function: results from the aging and longevity study in the Sirente geographic area (ilSIRENTE study) Gerontology 2006 52 24 32 10.1159/000089822 16439821

[B18] Bayliss EA Ellis JL Steiner JF Subjective assessments of comorbidity correlate with quality of life health outcomes: Initial validation of a comorbidity assessment instrument Health and Quality of life Outcomes 2005 3 51 10.1186/1477-7525-3-51 16137329 PMC1208932

[B19] O'Halloran J Miller GC Britt H Defining chronic conditions for primary care with ICPC-2 Fam Pract 2004 21 381 386 10.1093/fampra/cmh407 15249526

[B20] Galdas PM Cheater F Marshall P Men and health help-seeking behaviour: literature review J Adv Nurs 2005 49 616 623 10.1111/j.1365-2648.2004.03331.x 15737222

[B21] Katz JN Chang LC Sangha O Fossel AH Bates DW Can comorbidity be measured by questionnaire rather than medical record review? Medical Care 1996 34 73 84 10.1097/00005650-199601000-00006 8551813

[B22] Kriegsman DM Penninx BW van Eijk JT Boeke AJ Deeg DJ Self-reports and general practitioner information on the presence of chronic diseases in community dwelling elderly. A study on the accuracy of patients' self-reports and on determinants of inaccuracy J Clin Epidemiol 1996 49 1407 1417 10.1016/S0895-4356(96)00274-0 8970491

[B23] Klabunde CN Reeve BB Harlan LC Davis WW Potosky Am Do patients consistently report comorbid conditions over time?: results from the prostate cancer outcomes study Med Care 2005 43 391 400 10.1097/01.mlr.0000156851.80900.d1 15778642

[B24] Mukerji SS Duffy SA Fowler K Khan M Ronis DL Terrell JE Comorbidities in head and neck cancer: Agreement between self-report and chart review Otolaryngol Head Neck Surg 2007 136 536 542 10.1016/j.otohns.2006.10.041 17418247

[B25] Voaklander DC Kelly KD Jones A Suarez-Almazor ME Self Report Co-Morbidity and Health Related Quality of Life - A Comparison with Record Based Co-Morbidity Measures Social Indicators Research 2004 66 213 228 10.1023/B:SOCI.0000003554.03290.1a

[B26] Skinner KM Miller DR Lincoln E Lee A Kazis LE Concordance between respondent self-reports and medical records for chronic conditions: experience from the Veterans Health Study J Ambul Care Manage 2005 28 102 110 15923944 10.1097/00004479-200504000-00002

[B27] Gross R Bentur N Elhayany A Sherf M Epstein L The validity of self-reports on chronic disease: characteristics of underreporters and implications for the planning of services Public Health Reviews 1996 24 167 182 8918183

[B28] Corser W Sikorskii A Olomu A Stommel M Proden C Holmes-Rovner M Concordance between comorbidity data from patient self-report interviews and medical record documentation BMC Health Serv Res 2008 8 85 10.1186/1472-6963-8-85 18416841 PMC2346463

